# Supplementary material for: Zonisamide Administration Improves Fatty Acid β-Oxidation in Parkinson’s Disease
Source: Cells. 2018 Dec 29;8(1):14. doi: 10.3390/cells8010014 (PMC6356654; doi:10.3390/cells8010014)
Supplement: Supplementary file 1 [file cells-08-00014-s001.pdf]

Supplementary materials:

Zonisamide administration improves fatty acid β-oxidation in Parkinson's disease.

Shin-Ichi Ueno [1], Shinji Saiki [1,*], Motoki Fujimaki [1], Haruka Takeshige-Amano [1], Taku Hatano [1], Genko Oyama [1], Kei-Ichi Ishikawa [1,2], Akihiro Yamaguchi [2], Wado Akamatsu [2] and Nobutaka Hattori [1,*]

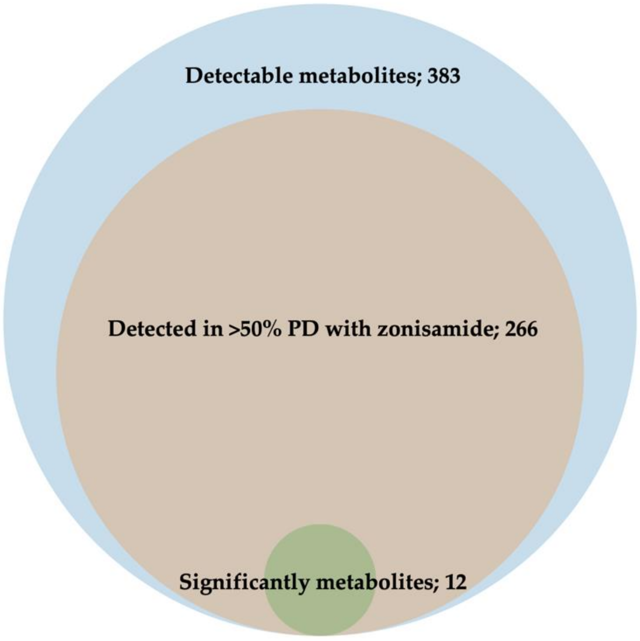

Supplementary Figure 1. Venn diagram showing the number of metabolites common to the experimental groups

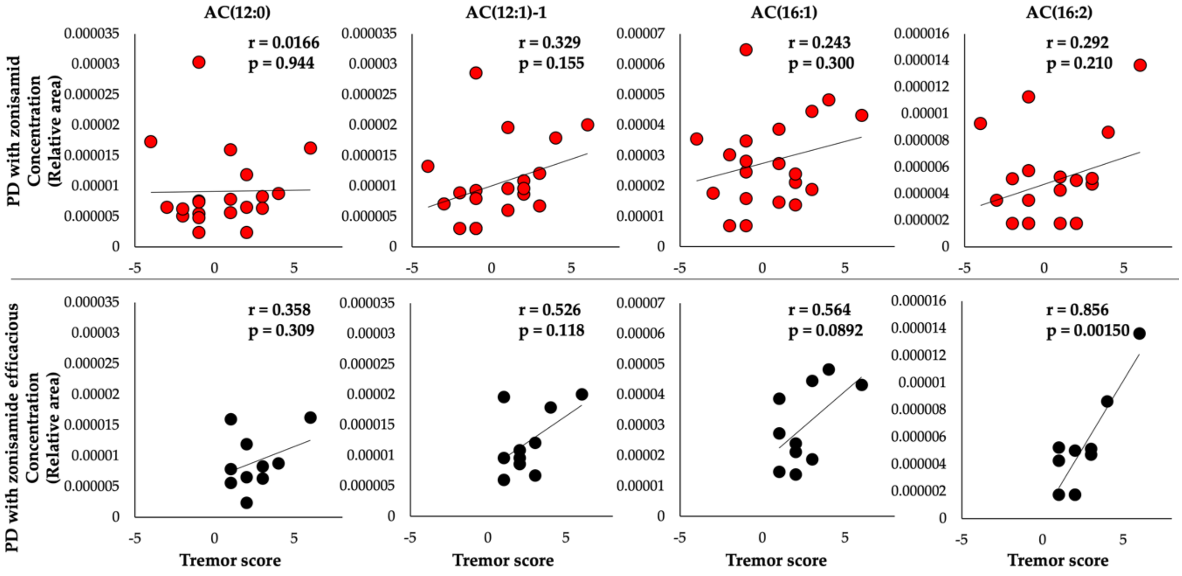

Supplementary Figure 2. Association of LCACs and tremor scores of UPDRS-III in motor amelioration of Parkinson's disease with zonisamide. Red indicates a correlation of tremor scores and LCACs in PD with zonisamide, while black indicates a correlation of tremor scores and LCACs in PD with efficacious zonisamide. Abbreviations: LCACs: long-chain acylcarnitines, PD: Parkinson's disease, UPDRS: Unified Parkinson's Disease Rating Scale. *p*-Value obtained by Spearman's rank correlation coeffecient.

Supplementary Table 1. Top 10 metabolites as factor loadings by partial least squares analysis.

Compound	R	*p*-Value
**1-methylnicotinamide**	0.467	0.00930
**Imidazolelactic acid**	0.430	0.0180
**XC0088**	0.413	0.0230
**AC(16:1)**	0.402	0.0280
**Stigmasterol**	0.395	0.0310
**XC0016**	0.382	0.0370
**Paraxanthine**	0.381	0.0380
**Ornithine**	0.376	0.0410
**AC(16:2)**	0.368	0.0460
**AC(12:1)-1**	0.359	0.0520

Abbreviations: XC0088, XC0016: unidentified metabolites annotated by Human Metabolome Technologies, Inc. AC: acylcarnitine. *p*-Value obtained by partial least squares regression analysis.

Supplementary Table 2. Long-chain acylcarnitines in Parkinson's disease with or without zonisamide.

Compound, relative area	Ratio of zonisamide (+) to zonisamide (-)	*p*-Value
**AC(12:0)**	1.73	0.0407
**AC(12:1)-1**	1.92	0.0405
**AC(12:1)-2**	1.66	<0.0001
**AC(12:1)-3**	1.01	0.0113
**AC(13:1)-1**	1.07	0.0142
**AC(13:1)-2**	1.25	0.708
**AC(14:0)**	1.54	0.320
**AC(14:1)**	1.54	0.708
**AC(14:2)-1**	1.50	0.179
**AC(14:2)-2**	-	-
**AC(14:3)**	-	-

**AC(15:0)**	1.00	0.912
**AC(16:1)**	1.75	0.0366
**AC(16:2)**	1.95	0.0054
**AC(18:0)**	1.27	0.281
**AC(18:1)**	1.38	0.165
**AC(18:2)-1**	1.27	0.165
**AC(18:2)-2**	-	-
**AC(20:0)**	-	-
**AC(20:1)**	1.27	0.322

Abbreviations: AC: acylcarnitine. *p*-Value obtained by Wilcoxon test.

Supplementary Table 3. The influence of zonisamide treatment duration and treatment dosage on the levels of four LCACs.

**Dependent variable**	**AC(12:0)**	**AC(12:1)-1**	**AC(16:1)**	**AC(16:2)**
	p-Value	*p*-Value	*p*-Value	*p*-Value
Treatment period	0.982	0.983	0.238	0.291
Zonisamide dosage	0.284	0.0940	0.633	0.937

Abbreviations: LCACs: long-chain acylcarnitines, *p*-Value obtained by multivariable regression analysis.

Supplementary Table 4. Serum skeletal-muscle-associated proteins in Parkinson's disease with or without zonisamide.

	**Zonisamide (-)**	**Zonisamide (+)**	***p*-Value**
**Creatine kinase [IU/L]<br>Mean (SD) [N.A.]**	223 (237)	104 (40.2) [2]	0.0798
**Aldolase [IU/L]<br>Mean (SD) [N.A.]**	4.49 (0.881)	3.68 (0.738) [2]	0.0344
**Creatinine [mg/dl]<br>Mean (SD)**	0.653 (0.141)	0.725 (0.256)	0.565
**HbA1c [%, NGSP]<br>Mean (SD)**	5.80 (0.258)	5.82 (0.474)	0.673

Abbreviations: IU/L: international unit per liter, SD: standard deviation, NGSP: National Glycohemoglobin Standardization Program. *p*-Value obtained by Wilcoxon test. N.A. = not available
